# Processing of Short-Form Content in Clinical Narratives: Systematic Scoping Review

**DOI:** 10.2196/57852

**Published:** 2024-09-26

**Authors:** Amila Kugic, Ingrid Martin, Luise Modersohn, Peter Pallaoro, Markus Kreuzthaler, Stefan Schulz, Martin Boeker

**Affiliations:** 1 Institute for Medical Informatics, Statistics and Documentation Medical University of Graz Graz Austria; 2 Institute for AI and Informatics in Medicine School of Medicine and Health Technical University of Munich Munich Germany

**Keywords:** electronic health records, EHR, clinical narratives, natural language processing, machine learning, deep learning, rule-based approach, short-form expression, disambiguation, word embedding, vector representations, language modeling, human-in-the-loop, feature extraction

## Abstract

**Background:**

Clinical narratives are essential components of electronic health records. The adoption of electronic health records has increased documentation time for hospital staff, leading to the use of abbreviations and acronyms more frequently. This brevity can potentially hinder comprehension for both professionals and patients.

**Objective:**

This review aims to provide an overview of the types of short forms found in clinical narratives, as well as the natural language processing (NLP) techniques used for their identification, expansion, and disambiguation.

**Methods:**

In the databases Web of Science, Embase, MEDLINE, EBMR (Evidence-Based Medicine Reviews), and ACL Anthology, publications that met the inclusion criteria were searched according to PRISMA (Preferred Reporting Items for Systematic Reviews and Meta-Analyses) guidelines for a systematic scoping review. Original, peer-reviewed publications focusing on short-form processing in human clinical narratives were included, covering the period from January 2018 to February 2023. Short-form types were extracted, and multidimensional research methodologies were assigned to each target objective (identification, expansion, and disambiguation). NLP study recommendations and study characteristics were systematically assigned occurrence rates for evaluation.

**Results:**

Out of a total of 6639 records, only 19 articles were included in the final analysis. Rule-based approaches were predominantly used for identifying short forms, while string similarity and vector representations were applied for expansion. Embeddings and deep learning approaches were used for disambiguation.

**Conclusions:**

The scope and types of what constitutes a clinical short form were often not explicitly defined by the authors. This lack of definition poses challenges for reproducibility and for determining whether specific methodologies are suitable for different types of short forms. Analysis of a subset of NLP recommendations for assessing quality and reproducibility revealed only partial adherence to these recommendations. Single-character abbreviations were underrepresented in studies on clinical narrative processing, as were investigations in languages other than English. Future research should focus on these 2 areas, and each paper should include descriptions of the types of content analyzed.

## Introduction

### Background and Significance

Clinical narratives, that is, free text authored by health professionals, are a core component of electronic health records (EHRs) within health care information systems. Today, findings reports, progress notes, surgery reports, and discharge summaries nearly seamlessly document the delivery of health care for every patient. However, with the adoption of EHRs, documentation times appear to increase for hospital staff [1-3], and this added time pressure often results in brevity in both documentation and communication. This explains the frequent use of short-form content (ie, abbreviations and acronyms) in routine documentation. While such jargon is generally well understood within a clinical specialty, professionals from other fields, and especially patients, often have to infer the meaning of these short forms in context. In-depth analyses of clinical narratives have highlighted the semantic and lexical ambiguities introduced by the use of short forms. This highlights the dilemma that, while clinical narratives present key information in a compact manner, overly cryptic formulations, if misunderstood, may severely impact patient safety [4-6].

This situation is further complicated when lexicon lookups for short forms yield multiple possible expansions. For example, the abbreviation “MS” in clinical narratives can stand for “morphine sulfate,” “multiple sclerosis,” or “mass spectrometry.” Determining the correct long form requires understanding the context. The complexity increases further with the ad hoc creation of many short forms and their use being restricted to particular institutions. The fact that the same short form can have entirely different meanings across medical specialties or health care institutions significantly influences the choice of processing methodology [4]. As a result of time pressures, clinicians rarely provide the long form alongside the first occurrence of the short form, as is customary in scientific publications and textbooks.

Natural language processing (NLP) has proposed several solutions to address this issue. The complexity of NLP applications varies across languages, depending not only on their grammatical and morphological characteristics (including those of clinical sublanguages) but also on the availability of lexical resources for each (sub)language. In most cases, low-resource languages have insufficient lexical coverage compared with high-resource languages, particularly English. The limited availability of clinical corpora for research is one reason many studies focus on similar data sets that have been released for research purposes after deidentification and ethics approval, such as Medical Information Mart for Intensive Care (MIMIC)-III [7].

In combination with NLP techniques, researchers have utilized existing methodologies to automatically identify, expand, and disambiguate short forms using data-driven shallow and deep learning (DL) approaches. However, current research does not clearly identify which methodologies are most effective in supporting short-form identification, expansion, and disambiguation. This scoping review, therefore, examines these 3 tasks, which represent distinct yet interconnected methodological aspects of each study, as they are closely linked to determining the correct long form for each short form.

This review covers a 5-year period and focuses on the narrative content of clinical data sets, the processing of short forms using state-of-the-art methodologies, and the short forms themselves. To the best of the authors’ knowledge, this is the first systematic scoping review to address these specific aspects of short-form processing in clinical narratives.

### Objective

This scoping review was conducted to identify relevant original research papers that apply NLP techniques to process short forms, such as acronyms and other abbreviations, in clinical narratives. All described methodologies need to be evaluated or validated in some form.

The objective of this review is to provide a systematic and structured overview of the literature on (1) short forms in clinical narratives and (2) methods used for their identification, expansion, and disambiguation.

## Methods

### Study Design

The study design adhered to the PRISMA (Preferred Reporting Items for Systematic Reviews and Meta-Analyses) guidelines [8] for conducting a systematic scoping review (Multimedia Appendix 1).

### Eligibility Criteria

Eligible articles were full-text, original, peer-reviewed publications that focused on machine learning (ML) or NLP techniques for the identification, expansion, or disambiguation of short-form content.

*Identification* focuses on detecting short-form content in clinical narratives, such as recognizing the acronym “RA” using the regular expression [A-Z]{2,}. *Expansion* involves generating possible long forms for short forms, such as expanding “RA” to “rheumatoid arthritis,” “right atrium,” or “room air.” Finally, *disambiguation* pertains to methods that determine the correct expansion for a short form. For example, in the context of “hypertension with RVSP of 46+ RA pressure,” the correct expansion is “right atrium” [9].

Papers under review were required to be written in either English or German, as all members of the review team are proficient in both languages. Additionally, the methodologies applied required each paper to focus on human clinical narratives, specifically textual content produced by clinicians in human medicine. This selection is based on the complex wording and structure of these narratives, which often include elements from different languages, such as Latin names for body parts or diseases, or untranslated foreign terms, adding complexity to text processing. Papers that did not focus on short forms were excluded.

Meta-analyses, case reports, collections, abstracts, surveys of patient-reported outcomes, papers lacking performance evaluation and validation, those that did not apply any ML or NLP methodology, and all types of reviews were excluded.

### Search Strategies

From January 1, 2018, to February 22, 2023, the literature databases Web of Science, Embase, MEDLINE, EBMR (Evidence-Based Medicine Reviews), and ACL Anthology were searched for relevant papers for this systematic scoping review. Web of Science was accessed through the official Clarivate website [10]. MEDLINE, Embase, and EBMR were searched via Ovid [11], while papers from ACL Anthology were retrieved using a custom Python (Python Foundation) search function, as the integrated ACL search does not support the operators NEAR or ADJ (adjacent). The search field operators assigned in each query, such as “TS” for “topic” or “.mp.” for “multiple purposes,” enable precise selection of papers from specific fields within the database records, thereby improving the accuracy and relevance of the search results. Examples of these fields are titles, abstracts, keywords, and subject headings. For ACL Anthology, a full-text search was conducted for all articles within the specified timeframe.

The search strategy primarily focused on short-form content and did not include “natural language processing” as a keyword, as this might have been too restrictive for capturing all relevant short-form content processing. Instead, the search terms were selected to cover all types of clinical narratives using MeSH (Medical Subject Headings) terms. Processing of any type of human clinical narrative was relevant to this scoping review. Table 1 presents a comprehensive list of search terms and strategies for each database, and the review process can be found in the *Results* section in a flow diagram.

**Table 1:** Search queries per database.

Database and query number		Search query
**Web of Science and ACL Anthology**		
	#1	TS=((medic* OR clinic* or allerg* OR androlog* OR anesthesiolog* OR anaesthesiolog* OR bariatric* OR biopharmaceutic* OR cardiolog* OR cardiovascul* OR chiropractic* OR cytopatholog* OR dental OR dentistr* OR dermatolog* OR dietetic* OR emergenc* OR endocrinolog* OR endodontic* OR ethnopharmacolog* OR forensic* OR gastroenterolog* OR genomic* OR geriatric* OR gerontolog* OR geroscienc* OR gynaecolog* OR gynecolog* OR haematolog* OR hematolog* OR immunolog* OR immunopatholog* OR microbiolog* OR midwife* OR nanomedic* OR neonatolog* OR nephrolog* OR neurolog* OR neuropatholog* OR neuropharmacolog* OR neuropsychiatr* OR neuroradiolog* OR neurosurg* OR neurotolog* OR nursing OR nutrigenomic* OR obstetric* OR occupational* OR oncolog* OR ophthalmolog* OR optometr* OR orthodontic* OR orthopedic* OR orthoptic* OR otolaryngolog* OR otolog* OR otorhinolaryngolog* OR paramedic* OR patholog* OR pediatric* OR perinatolog* OR periodontic* OR pharmacogenetic* OR pharmacolog* OR pneumolog* OR pneumonolog* OR podiatr* OR proctolog* OR prosthodontic* OR psychiatr* OR psychopharmacolog* OR pulmonolog* OR radiolog* OR radiology* OR rehabilitati* OR rheumatolog* OR surgery OR surgic* OR telemedic* OR telepatholog* OR teleradiolog* OR telerehabil* OR toxicolog* OR traumatolog* OR urolog* OR venereolog*) NEAR/4 (text* or narrati* or document* or summar* or note* or report*) )
	#2	TS=(abbrev* OR acronym* OR ( short* NEAR/2 form* ) OR ( (single OR two OR three OR four) NEAR/2 (character or characters)) OR ellips* OR initialism*)
	#3	DOP=(2018-01-01/2023-02-22)
	#4^a^	#1 AND #2 AND #3
**MEDLINE, Embase, and all EBMR^b^ reviews**		
	#1	((medic* OR clinic* or allerg* OR androlog* OR anesthesiolog* OR anaesthesiolog* OR bariatric* OR biopharmaceutic* OR cardiolog* OR cardiovascul* OR chiropractic* OR cytopatholog* OR dental OR dentistr* OR dermatolog* OR dietetic* OR emergenc* OR endocrinolog* OR endodontic* OR ethnopharmacolog* OR forensic* OR gastroenterolog* OR genomic* OR geriatric* OR gerontolog* OR geroscienc* OR gynaecolog* OR gynecolog* OR haematolog* OR hematolog* OR immunolog* OR immunopatholog* OR microbiolog* OR midwife* OR nanomedic* OR neonatolog* OR nephrolog* OR neurolog* OR neuropatholog* OR neuropharmacolog* OR neuropsychiatr* OR neuroradiolog* OR neurosurg* OR neurotolog* OR nursing OR nutrigenomic* OR obstetric* OR occupational* OR oncolog* OR ophthalmolog* OR optometr* OR orthodontic* OR orthopedic* OR orthoptic* OR otolaryngolog* OR otolog* OR otorhinolaryngolog* OR paramedic* OR patholog* OR pediatric* OR perinatolog* OR periodontic* OR pharmacogenetic* OR pharmacolog* OR pneumolog* OR pneumonolog* OR podiatr* OR proctolog* OR prosthodontic* OR psychiatr* OR psychopharmacolog* OR pulmonolog* OR radiolog* OR radiology* OR rehabilitati* OR rheumatolog* OR surgery OR surgic* OR telemedic* OR telepatholog* OR teleradiolog* OR telerehabil* OR toxicolog* OR traumatolog* OR urolog* OR venereolog*) adj4 (text* or narrati* or document* or summar* or note* or report*) ).mp.
	#2	(abbrev* OR acronym* OR ( short* adj2 form* ) OR ( (single OR two OR three OR four) adj2 (character or characters)) OR ellips* OR initialism*).mp.
	#3	#1 AND #2
	#4^a^	limit 3 to yr=”2018-2023”

^a^Query number #4 is the final applied query, which incorporates all other queries #1, #2, and #3.

^b^EBMR: Evidence-Based Medicine Reviews.

### Selection of Studies

For the scoping review, database records were imported into Citavi (Swiss Academic Software GmbH) [12], version 6. During the deduplication stage, these records were reviewed independently by 4 team members (AK, IM, LM, and PP). Each team member was assigned a portion of the records for deduplication: IM, LM, and PP each reviewed 20%, while AK reviewed 40%. After deduplication, AK conducted the merging and final review of all papers. In the screening stage, 4 team members were assigned portions of records to screen titles and abstracts for eligibility. Papers marked for exclusion were moved to a separate directory in Citavi. Each directory was reviewed for validation by at least one supervisor from the review team (MB, MK, or SS). In the eligibility stage, full-text papers were reviewed by 4 team members in pairs (AK and PP/IM and LM). If any team identified reports that matched the exclusion criteria, these were reviewed again by AK and one of the 3 supervisors. Any disagreements were discussed with additional team members until a consensus was reached.

### Data Extraction

After the final inclusion decision, all eligible papers were read by all team members to validate the extracted information, which was recorded in a Google Spreadsheet (Alphabet Inc./Xxvi Holdings Inc.). The extracted data included the following information: publication title, authors, year of publication, short task description, setting (where the study was performed), study type, data set description, data set language, type of clinical narrative, scope of clinical narrative, study population, NLP methodologies, experimental setup, benchmarks or ground truth or gold standard, baselines, evaluation and validation, results, performance metrics, limitations, and conceptual description of short forms.

### Data Synthesis

Publications were categorized based on (1) the type of short forms being processed and (2) the research methodology. For each type of short form, all processed data sets, data set descriptions, preprocessing methods, and examples provided in the full-text articles were synthesized to determine if any restrictions were placed on the data set before processing. This involved allocating specific restrictions for data set processing, from which short-form types could be derived, or determining if no restrictions were applied. For categorizing research methodologies, the 3 target objectives for short-form processing (identification, expansion, and disambiguation) were extracted from each publication, along with assigning multidimensional research methodologies to each objective. Additional objectives included differentiating between languages studied, noting NLP study recommendations, and identifying applied data sets, inspired by the scoping review by Kersloot et al [13]. Information was extracted from each article, and the characteristics were assigned accordingly. Overall occurrence rates were used for narrative description and summarization.

## Results

### Overview

**Figure 1:**
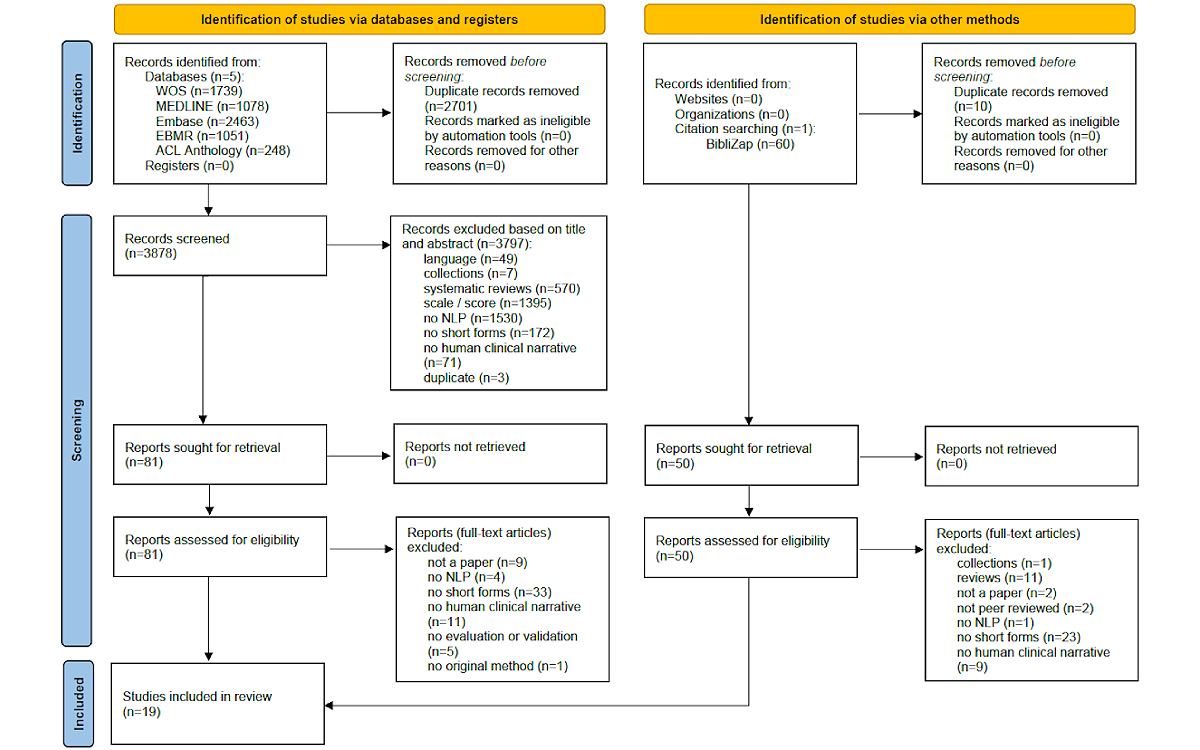
PRISMA diagram for systematic scoping review. EBMR: Evidence-Based Medicine Reviews; NLP: natural language processing; PRISMA: Preferred Reporting Items for Systematic Reviews and Meta-Analyses; WOS: Web of Science.

Figure 1 illustrates the workflow of this scoping review. A total of 6579 records were identified that matched the search criteria via literature databases. After deduplication, 3878 records proceeded to the screening stage. Reviewing titles and abstracts reduced this number to 81 papers, which were then assessed for eligibility by reading their full texts. An additional manual citation search was conducted using BibliZap [14] based on the included studies, yielding 50 more full-text articles for eligibility screening. A thorough analysis during the eligibility stage resulted in 19 articles being included in the final analysis.

The results were divided into 5 sections, covering (1) types and processing of short forms, (2) applied data sets, (3) languages under investigation, (4) adherence to NLP recommendations, and (5) overall findings.

### Types of Short Forms

The exact type of short-form content was not explicitly stated in all included articles. It had to be extracted through examples provided, data set evaluations and descriptions, or inference. Unfortunately, a clear typology of short forms does not exist, leading to varying definitions and interpretations of short-form types depending on the data set language used in each article.

In this scoping review, the term “short form” is used as a general category for all types of abbreviations. It includes not only classical acronyms or initialisms, such as “ECG,” “5-FU,” and “AIDS,” but also all types of abbreviations, including single-character abbreviations such as “N” for “nerve” or “neoplasm.” Additionally, short forms encompass unfinished words, also known as ad hoc abbreviations, such as “pat” for “patient” or “neonat” for “neonatology.” In contrast to acronyms, these short forms are not capitalized and, depending on the language, may end with a dot, for example, “chron.” (chronic) or “Dr.” (doctor).

Acronyms, such as “RA” for “rheumatoid arthritis,” are terms for shortened words or phrases that are written in uppercase letters and are defined based on their pronunciation. However, the inclusion of these acronyms in NLP tasks cannot always be guaranteed, as articles may define short forms in a manner that provides reliable results only for a subset of the data set. For example, only acronyms of 3 characters in length may be processed and evaluated.

Additionally, the occurrence of specialized abbreviations varies depending on the data set language. For example, single-character abbreviations, such as “A” for “arteria” or “aorta,” are common in German clinical narratives but less frequent in other languages, such as English. Uppercase letters followed by a period are prevalent in German, such as “M.” for “muscle” or “morbus” (disease), and “V.” for “vein” or “vulnus” (wound). By contrast, English clinical narratives often use uppercase letters followed by “x,” such as “Hx,” “Dx,” and “Fx” for medical history, diagnosis, and fracture.

### Data Set Restrictions

Any restrictions placed on the data set before processing short forms are crucial for evaluating the effectiveness of methodologies for different types of short forms. For example, a restriction might involve analyzing only acronyms within a data set and excluding all other types of short forms.

As many as 7 of the 19 (37%) included papers [15-21] had no restrictions regarding the evaluation of short forms, meaning their investigation of identification, expansion, and disambiguation was not preemptively focused on specific types of short forms. By contrast, the majority (12/19, 63%) of papers [22-33] did impose restrictions on the type of short form, either through length restrictions, such as rule-based filters, or preprocessing guidelines. For example, regarding single-character abbreviations, 1 (5%) article [28] included only a few such abbreviations while excluding others. In 2 (11%) papers [23,26], the inclusion or exclusion of single-character abbreviations was unclear and could not be determined. Table 2 provides a full list of short-form types and the restrictions placed on data sets.

**Table 2:** Restrictions placed on the data set and selection of types of short forms before being processed.

Paper reference	Restrictions on short forms	Types of short forms selected
Adams et al [22]	Yes	Acronyms
Avdic et al [23]	Yes	Shortened words, abbreviations followed by a period character, physician-specific abbreviations, and common abbreviations
Egorov and Funkner [24]	Yes	Acronyms and abbreviations (at least half of the letters of a word need to be uppercase to be defined as an abbreviation)
Jaber and Martínez [30]	Yes	Acronyms and abbreviations (defined as a short form of a word or phrase)
Jaber and Martínez [25]	Yes	Acronyms and abbreviations (defined as a short form of a word or phrase)
Luo et al [15]	No	N/A^a^
Skreta et al [26]	Yes	Acronyms and abbreviations (without consideration of the casing of the word, all in lowercase)
Grossman et al [16]	No	N/A
Grossman Liu et al [17]	No	N/A
Wang et al [18]	No	N/A
Link et al [27]	Yes	Acronyms: 2 characters in length
Khaleghi et al [28]	Yes	Abbreviations (mostly user-specific word truncation): length less than 3 characters excluded, but hand-selection and inclusion of 2 single-character and 2 two-character abbreviations
Kashyap et al [29]	Yes	Acronyms: more than 2 characters in length
Joopudi et al [19]	No	N/A
Rajkomar et al [20]	No	N/A
Mykowiecka and Marciniak [31]	Yes	Ad hoc abbreviations
Báez et al [21]	No	N/A
Seneviratne et al [32]	Yes	Acronyms
Agrawal et al [33]	Yes	Acronyms

^a^N/A: not applicable.

### Processing of Short Forms

Various methods are applied to process clinical narratives across the 3 steps: identification, expansion, and disambiguation of short forms. The first section provides an overview of all methodological classes and their functionalities found in the articles. The second section discusses the methods applied to each of the 3 processing steps.

### Overview of Methodologies

The methods described in the selected articles can be roughly categorized into 8 classes: handcrafted rules, classical ML, DL, language modeling, human-in-the-loop, text representations, feature extraction, and software packages. Table 3 presents the classification of methodologies for processing short forms. Predominantly, classical ML techniques are used in combination with handcrafted rules for short-form processing, followed by applications of DL and language modeling. The human-in-the-loop approach is mentioned in only 1 article [18].

**Table 3:** Methods used for short-form processing.

Paper reference	Handcrafted rules	Classical machine learning	Deep learning	Language modeling	Human-in-the-loop	Text representations	Feature extractions	Software packages
Adams et al [22]	N/A^a^	N/A	✓	✓	N/A	✓	N/A	N/A
Avdic et al [23]	✓	✓	N/A	N/A	N/A	N/A	N/A	N/A
Egorov and Funkner [24]	✓	✓	N/A	N/A	N/A	✓	N/A	N/A
Jaber and Martínez [30]	N/A	✓	N/A	N/A	N/A	✓	✓	N/A
Jaber and Martínez [25]	N/A	N/A	✓	✓	N/A	✓	N/A	N/A
Luo et al [15]	✓	✓	✓	✓	N/A	✓	✓	✓
Skreta et al [26]	N/A	✓	N/A	N/A	N/A	✓	N/A	N/A
Grossman et al [16]	✓	N/A	N/A	N/A	N/A	N/A	✓	✓
Grossman Liu et al [17]	✓	✓	✓	✓	N/A	N/A	✓	✓
Wang et al [18]	N/A	✓	N/A	N/A	✓	N/A	✓	✓
Link et al [27]	✓	✓	N/A	N/A	N/A	✓	N/A	✓
Khaleghi et al [28]	✓	✓	N/A	N/A	N/A	✓	✓	N/A
Kashyap et al [29]	✓	✓	N/A	N/A	N/A	N/A	N/A	N/A
Joopudi et al [19]	✓	✓	✓	N/A	N/A	✓	✓	N/A
Rajkomar et al [20]	✓	✓	✓	✓	N/A	N/A	✓	N/A
Mykowiecka and Marciniak [31]	✓	N/A	✓	N/A	N/A	✓	✓	✓
Báez et al [21]	N/A	✓	✓	N/A	N/A	✓	N/A	✓
Seneviratne et al [32]	✓	N/A	✓	✓	N/A	✓	N/A	N/A
Agrawal et al [33]	✓	N/A	✓	✓	N/A	N/A	N/A	N/A

^a^N/A: not applicable.

*Handcrafted rules* involve the manual implementation of rule-based approaches, such as regular expressions, for specific tasks during text processing. For example, a regular expression might be used to identify acronyms in clinical texts.

*Classical ML* methods primarily involve supervised statistical modeling techniques, including support vector machines (SVMs), naïve Bayes classifiers, and decision tree classifiers, as well as unsupervised clustering approaches. These methods are used to classify data points into distinct categories, whether they are seen or new.

*Statistical modeling techniques*, such as conditional random fields [34], hidden Markov models [35], and logistic regression [36], use statistical functions to learn from the data set and generate predictions based on given dependencies. For instance, logistic regression performs binary classification by applying weights, a sigmoid function, and probabilistic interpretation to train a model for the classification task.

*SVMs* [37] map data points to a feature space to find the best hyperplane that separates different classes in the data set. Support vectors are the data points closest to this hyperplane. SVMs aim to maximize the margin between support vectors, which enhances the model’s performance on unseen data and makes it more reliable and robust to irregularities or outliers in the data set.

*Naive Bayes* [38] is a probabilistic classifier based on Bayes’ theorem, with the “naive” assumption that features are independent of each other. It calculates the probability of existing features to determine the likelihood that unseen features belong to predetermined groups.

*Decision tree classifiers* [39], including methods such as random forest [40] and gradient boosting [41], use a tree structure with nodes and branches to represent decisions based on features. Internal nodes represent decisions based on feature sets, branches represent possible outcomes of those decisions, and leaf nodes represent the final predictions. This tree structure facilitates traceable and interpretable classifications, allowing the decision path to be traced back to the root node, which aids in the explainability of the model.

*DL* methods, such as recurrent neural networks, convolutional neural networks (CNNs), sequence-based models, and transfer learning, use artificial neural networks with multiple layers of interconnected nodes. Four key features distinguish DL from classical ML: feature engineering, model complexity, data requirements, and interpretability. In classical ML, features are typically hand-selected or integrated through domain knowledge by engineers, and the trained models have simpler structures. With fewer features, classical ML models need less data and offer more interpretable classifications. By contrast, DL models automatically extract complex features from data sets, reducing the need for manual feature engineering. These models are more complex due to their multiple layers of interconnected neurons and a larger number of parameters. As a result, DL models require larger data sets and more resources for training compared with classical ML models. Additionally, DL models are often considered “black boxes” and lack interpretability.

*Language modeling* is closely related to DL. It encompasses techniques that use pretrained language models created from large corpora of text using various NLP algorithms, such as Bidirectional Encoder Representations from Transformers (BERT) [42] and Efficiently Learning an Encoder that Classifies Token Replacements Accurately (ELECTRA) [43]. These models use methods such as masked language modeling and bidirectional encoders. Fine-tuning or adapting pretrained language models to specific problem domains with domain-specific data sets can further enhance their performance for NLP tasks, such as classification or prediction. The purpose of language modeling is to capture the statistical properties of language to improve the contextual understanding of texts. A recent advancement in this field is the development of large language models (LLMs), such as generative pretrained transformers (GPTs) [44,45]. A prominent example is ChatGPT [46], which encompasses GPT models developed by OpenAI that generate and synthesize text based on input prompts.

*Human-in-the-loop* methods [47] integrate human expertise or feedback into the ML process, applicable in both classical ML and DL settings. Examples are active learning, where human input guides the selection of labels or features during training; and model evaluation, where human feedback is used to interpret predictions and improve the model.

*Text representations* involve transforming textual input before data processing, significantly impacting overall performance depending on the chosen methodology. Examples are bag-of-words, word embeddings, and n-grams.

*Bag-of-words* represents text as a collection of individual words, disregarding grammar and word order. In this approach, only the presence of words is considered, while word sequences and sentence structure are ignored.

*Word embeddings* are vector representations of words in a continuous vector space, capturing the similarity between words based on their context and meaning. Each word is represented as a vector with a fixed dimension, and words with similar meanings or contexts are positioned close together in the vector space. This representation preserves semantic information and the context of the input.

*N-grams* are contiguous sequences of *n* items (or words) from a text. The input text is divided into consecutive sequences of *n* items, which can be words, characters, or similar structures. This representation scheme captures the word order and context within the text.

*Feature extraction* methods involve extracting or calculating features from text segments. Examples are string similarity metrics, such as those introduced by Levenshtein [48] or Needleman and Wunsch [49], part-of-speech tagging, and sectioning clinical narratives using section header information.

*Software packages* include implemented techniques and algorithms used by authors either as a baseline or integrated into their own workflow for short-form processing.

### Methodologies for Identification, Expansion, and Disambiguation

Table 4 describes the methods for processing short forms, including identification, expansion, or disambiguation. Because of the variability in the objectives of each paper, not all papers addressing expansion also cover disambiguation, and not all papers focusing on disambiguation address identification or expansion of short-form content.

In our review, 14 of the 19 (74%) articles apply methods for identification, 12 (63%) for expansion, and 15 (79%) for disambiguation of short-form content. Of these, only 2 of 14 (14%) identification methods, 2 of 12 (17%) expansion methods, and 15 of 15 (100%) disambiguation methods provide performance metrics.

**Table 4:** Methodologies for the identification, expansion, and disambiguation of short forms.

Paper reference	Identification	Expansion	Disambiguation
Adams et al [22]	N/A^a^	N/A	Gaussian embedding drawn from both word and metadata prior densities, Bayesian skip-gram model, variational distribution over latent meaning cell, and deep learning (BERT^b^, embeddings from language models)
Avdic et al [23]	Rules, lookup in custom lexical resource, baselines, support vector machine, random forest, tree tagger, naive Bayes, and variations of methodologies	N/A	N/A
Egorov and Funkner [24]	Rules and lookup in custom lexical resource	Bag-of-characters vectors, 2 TF-IDF^c^ vectorizers	Random forest, logistic regression, extreme gradient boosting, stochastic gradient descent, CatBoost (gradient boosting on decision trees), and support vector classification
Jaber and Martínez [30]	N/A	N/A	Feature extraction, embedding vectors utilized from pretrained models, support vector machine, and naive Bayes skip-gram models
Jaber and Martínez [25]	N/A	N/A	BERT language models, no fine-tuning, context and expansions fed into models as input, token-type IDs, and binary mark IDs
Luo et al [15]	Rules, string similarity (Levenshtein [48]), regular expressions	Lookup and ranking with machine learning	Feature vectors, cosine distances, edit distances, similarity, and plus combinations
Skreta et al [26]	N/A	N/A	Convolutional neural network and max pool over time for local context, IDF^d^-weighted embedding average for global context, and combination of both outputs
Grossman et al [16]	CARD^e^ framework [50] (combination of regular expressions and string similarity approaches)	Harmonization of different repositories into 1, cross-mapping synonymous record with filtering, string similarity, and MetaMap [51]	N/A
Grossman Liu et al [17]	CARD framework [50] (combination of regular expressions and string similarity approaches)	Harmonization of different repositories into 1, cross-mapping synonymous record with string similarity, rule-based text feature replacement, and meta inventory potential pair selection	Feed-forward dense neural network, gradient-boosted model, and transformer model
Wang et al [18]	Interactive learning, human annotator input (context + sense, feature + sense)	Dynamic feature representation combined with parameter estimation (logistic regression) = probability predictions	Instance selection is shown to the human annotator if the classifier is unable to select a sense, compared with random sampling, active learning, ReQuery-ReClassify expert method (Wang et al [52]), and feature engineering
Link et al [27]	Rule-based selection of notes with acronyms	Random forest for noisy labels, target sense classification, and word embeddings	Averaging predicted probabilities for final probability selection, prevalence estimation with probability cut-off, and baselines include most-frequent sense and knowledge-based method (Finley et al [53])
Khaleghi et al [28]	Regular expressions (filtering), lemmatization, tokenization, stemming, and string similarity (Levenshtein [48])	String similarity Levenshtein matrix, plus updated matrix, hierarchical agglomerative clustering, empirical selection of cut-off distance, heuristic clustering—sorting the hierarchical agglomerative clustering based on intercluster distances	N/A
Kashyap et al [29]	Regular expressions	Lookup in the PubMed database for text and possible expansions	Logistic regression model
Joopudi et al [19]	Regular expressions	String matching, feature vectors for each entity (bag-of-words, part-of-speech, clinical note, section, n-gram), assignment of each abbreviation to appropriate cluster, and proportional sampling of sentences from all clusters	Support vector machine, convolutional neural network, and baseline including most-frequent sense
Rajkomar et al [20]	Regular expressions	Needleman and Wunsch [49] global sequence alignment (token level)	Reverse substitution of abbreviations on web data, transfer learning, and chained inference technique to overcome domain shift
Mykowiecka and Marciniak [31]	Rules, part-of-speech tagger (Concraft2 [54]), and morphologic analyzer (Morfeuz2 [55])	Lookup in self-created resources generated through rules, clustering via the Chinese Whispers algorithm [56], and cosine similarity	Word2vec models, bidirectional long-short term memory, and baseline including most-frequent sense
Báez et al [21]	Named entity recognition via Flair framework (bidirectional long-short term memory-conditional random fields architecture) and application of clinical word embeddings	N/A	N/A
Seneviratne et al [32]	Rules	Lookup, replacement of acronyms in sentences with possible expansions to create sentence embeddings	Triplet networks, triplet loss, modeled as a binary classification problem, and baseline including acronym span prediction via pretrained language models (SciBERT and BioBERT)
Agrawal et al [33]	N/A	N/A	Generative pretrained transformer GPT^f^-3 as LLM^g^; LLM prompting for the resolution, postprocessing LLM answer with rules, weak supervision with data set filtering, and fine-tuned PubMedBERT [57] model for evaluation of output

^a^N/A: not applicable.

^b^BERT: bidirectional encoder representations from transformers.

^c^TF-IDF: term frequency-inverse document frequency.

^d^IDF: inverse document frequency.

^e^CARD: Clinical Abbreviation Recognition and Disambiguation.

^f^GPT: generative pretrained transformer.

^g^LLM: large language model.

### Identification

The most common NLP approaches for short-form identification (12/14, 86%) include rules and regular expressions, lookups in lexical resources (either custom-created or freely available), and string similarity calculations. Lemmatization, tokenization, and stemming combined with rules were used only by Khaleghi et al [28]. By contrast, Wang et al [18] applied a supervised method, incorporating human annotation input to develop a logistic regression model, rather than using these NLP techniques. Similarly, Mykowiecka and Marciniak [31] used rules in combination with part-of-speech taggers and morphological analyzers to identify ad hoc abbreviations, such as unfinished words, in Polish clinical narratives.

Performance metrics for short-form identification were reported by only 2 studies. Avdic et al [23] conducted the identification and labeling of terms in their Serbian clinical corpus, where 12.9% of the words were abbreviations. By using normalization, stemming, cut-offs, and custom dictionaries, they improved the labeling of Serbian medical terms (including diagnoses, symptoms, medications, etc) to achieve an *F*_1_-score of 0.908.

Báez et al [21] developed the Chilean Waiting List Corpus, an annotated resource comprising deidentified physician-authored referrals from various clinical specialties in Spanish. The annotations include mentions of findings, procedures, diseases, medications, body parts, and abbreviations. Using this annotated data set, they implemented a named entity recognition model with the Flair framework [58], which generated contextual embeddings for each word. Pretrained embeddings, trained on Spanish Wikipedia articles, were compared with embeddings enhanced with clinical data from unannotated parts of the corpus. An abbreviation detection model created with this approach achieved an *F*_1_-score of 0.92 for both the base and enhanced pretrained embedding versions.

### Expansion

The expansion of short forms can be achieved either through lookup operations or nonlookup methods. Lookup-based expansion involves searching for a short form in a list, corpus, sense inventory, or dictionary to retrieve possible expansion candidates. These candidates can then be processed further using other methodologies for disambiguation. Nonlookup expansion methods include data mining techniques, end-to-end encoder-decoder models, text generation workflows, and active learning approaches. Expansion of short forms is accomplished through lookup operations in 8 of 12 (67%) articles [16,17,19,24,27,29,31,32]. These methods involve searching for short forms in lists, corpora, or dictionaries to find possible expansions. By contrast, 3 of 12 (25%) articles used nonlookup methods [18,20,28], which do not rely on predefined lists or dictionaries. Additionally, 1 article (1/12, 8%) by Luo et al [15] presented results from the n2c2 challenge, where participating teams used a mix of lookup operations, such as semantic-type classifiers, vocabulary classifiers, and similarity scores, as well as nonlookup methods, such as edit distance calculations with word embeddings.

Most of these approaches incorporate a variety of methodologies, including feature vector creation and representation (such as a bag of words, part of speech tagging, clinical notes, section information, and n-grams), logistic regression, random forest, word embeddings, retrieve and rank approaches, and clustering. Notably, Rajkomar et al [20] applied Needleman-Wunsch global sequence alignment, a method originally developed for nucleotide and protein sequence alignment, to clinical texts and abbreviation expansion.

Mykowiecka and Marciniak [31] generated expansion candidates either through a data-driven rule-based approach derived from the clinical texts or by applying the Chinese Whispers algorithm [56]. This randomized graph-clustering method clusters occurrences of abbreviations and identifies expansion candidates based on these clusters and cosine similarity.

Grossman et al [16] developed an extensive database of medical short forms, termed the “metathesaurus” by consolidating various repositories. Their processing techniques included lexical normalization, concept identification using MetaMap [59], and cross-mapping of synonymous terms through string similarity. Coverage calculations indicated that their resource achieved very high micro coverage, with 94.3% short-form coverage and 99.6% sense coverage, significantly outperforming the UMLS (Unified Medical Language System) LRABR acronym-abbreviation table, which covered only 74.8% of short forms.

Khaleghi et al [28] utilized similarity matrix calculations using Levenshtein distance [48,60], incorporating rule-based adjustments based on the type of short form processed. The results were then input into k-means partitional clustering, with empirical cut-off distances set and clusters sorted based on intercluster distances. This approach achieved an abbreviation detection accuracy of 90% and a typo detection accuracy of 90.6%.

### Disambiguation

The disambiguation approaches are predominantly based on DL methods, which account for 8 of the 15 (53%) studies, including CNNs, feed-forward neural networks, and transformers. Classical ML methods make up 3 of the 15 studies (20%), with SVMs being a notable example. Additionally, embedding representations, such as Gaussian and cosine distance, are used in 6 of the 15 (40%) studies. Logistic regression was used in 2 of the 15 (13%) cases. Other methodologies include Bayesian skip-gram models, variational distributions, gradient boosting, and chained inference combined with transfer learning, among other techniques.

According to performance measures reported by Egorov and Funkner [24], SVM emerged as the best model for short-form expansion, achieving an *F*_1_-score of 0.937. Their study compared SVM with other methods, including random forest, logistic regression, gradient boosting, and stochastic gradient descent classifiers, with the classical ML method outperforming the others in this context.

Adams et al [22] utilized contextualized word representations derived from local context and metadata, combined with predefined inventories of short-form expansions. They used Gaussian embeddings drawn jointly from word and metadata prior densities, and a Bayesian skip-gram model to process surrounding words. This approach resulted in a variational distribution over the latent meaning cell, surpassing the performance of DL strategies. It achieved a weighted mean *F*_1_-score across 5 pretraining runs of 0.69 for MIMIC-III, 0.57 for the CUIMC (Columbia University Irving Medical Center) data set, and 0.51 for the Clinical Abbreviation Sense Inventory (CASI) [9] data set from the University of Minnesota.

Joopudi et al [19] combined string matching, a custom word-sense inventory, and deep-learning methods with feature vectors for SVM and CNN. Among these, CNN with local features outperformed SVM by incorporating clinical narrative metadata and section information, achieving a micro-averaged accuracy of 0.979.

Jaber and Martínez [30] analyzed a subset of 13 acronyms from the CASI data set for acronym disambiguation using SVM and naive Bayes skip-gram models. During the training phase, feature extraction and embedding vectors were utilized alongside pretrained skip-gram models from PubMed Central, Wikipedia, and PubMed abstracts. SVM outperformed naive Bayes, achieving an average accuracy of 0.97 compared with 0.93.

In a follow-up study, Jaber and Martínez [25] utilized a masked language modeling approach with 3 pretrained BERT [42] language models, without fine-tuning for the specific problem domain. They incorporated the context and expansions of each short form as input to the model. This approach achieved an accuracy of 0.991, surpassing the results reported by Adams et al [22] and Joopudi et al [19] on the CASI data set.

Luo et al [15] detailed the methodologies of the top 10 performing teams in the shared task on clinical concept normalization. For the challenging category of single-character abbreviations, 2 teams distinguished themselves by using DL with contextual embeddings. Despite achieving a maximum accuracy of only 0.35, their performance surpassed that of most other teams.

Skreta et al [26] used UMLS term embeddings combined with reverse substitution of terms (replacing expansions with their corresponding abbreviations) in the MIMIC-III data set to generate training examples. By leveraging concept hierarchies from UMLS to augment training sets and searching for related concepts, they adapted the global context in narratives using Euclidean distance to develop an abbreviation disambiguation pipeline with CNNs. By integrating concept hierarchies during pretraining, augmenting with associated medical concepts extracted from the embedding space, and considering the global context of clinical narratives, Skreta et al [26] achieved an accuracy of 0.841 on the CASI data set with their CNN-based abbreviation disambiguation pipeline.

Grossman Liu et al [17] continued the work of Grossman et al [16] by applying DL methodologies to cross-map short forms and develop a metathesaurus of clinical short forms. On clinician-labeled data, their gradient-boosted model, BERT model, and an ensemble approach demonstrated similar performance, with the ensemble achieving an *F*_1_-score of 0.814.

Wang et al [18] utilized an active learning algorithm, incorporating human annotator input to enhance acronym disambiguation. This approach achieved an area under the learning curve score of 0.852 on the CASI data set.

Link et al [27] assessed their semisupervised ensemble ML algorithm (CASEml) on the acronyms “RA,” “MS,” and “MI” using Veterans Affairs EHR data. Their approach, which integrated a visit-level random forest with embeddings-based context representation, achieved accuracy metrics of 0.947 for “RA,” 0.911 for “MS,” and 0.706 for “MI.”

Kashyap et al [29] developed CLASSE GATOR (Clinical Acronym Sense Disambiguator) for acronym disambiguation. The tool, which utilizes full-text research articles from PubMed Central to detect and extract acronym-expansion pairs, trained a logistic regression model. The system achieved an average accuracy of 0.879 in predictive performance.

Rajkomar et al [20] developed a single translation model for the detection, expansion, and disambiguation of clinical acronyms, and evaluated it on multiple data sets. The model achieved accuracies of 0.921 on the CASI data set, 0.957 on the MIMIC-III data set, and 0.965 on the Informatics for Integrating Biology and the Bedside (i2b2) 2014 data set.

Mykowiecka and Marciniak [31] used a bidirectional long short-term memory network architecture for disambiguating ad hoc abbreviations, achieving an *F*_1_-score of 0.726 with 10-fold cross-validation. Further variations in the lists of possible expansion candidates improved the *F*_1_-score to 0.968.

Seneviratne et al [32] adapted embeddings-based approaches for acronym disambiguation by learning sentence embeddings to capture semantic differences. They applied triplet networks and triplet loss methods, drawing inspiration from Siamese Networks [61] for image recognition and triplet neural networks [62] for predicting protein gene ontology. To create the embeddings, acronyms were identified using rules, and based on the CASI data set, abbreviations were replaced with their possible long forms. This approach was modeled as a binary classification problem, where the method determines the correctness of an acronym by comparing the input with the trained embeddings, achieving an *F*_1_-score of 0.87.

Agrawal et al [33] tested the disambiguation of acronyms from the CASI data set using GPT-3, a GPT model developed by OpenAI. This LLM utilizes contextual understanding from the input prompt to generate appropriate responses. In this case, no specific examples for acronym resolution were provided; instead, the model was given only the context in which the acronym appeared, followed by a request to expand the acronym based on that context. This method achieved 0.86 in accuracy and 0.69 in macro *F*_1_-score. With additional data set filtering and fine-tuning of PubMedBERT [57] to distill the GPT-3 model into a smaller, more manageable version, evaluations on MIMIC—restricted for GPT-3 due to data-use agreements—were conducted. The combined GPT-3 and PubMedBERT approach achieved an accuracy of 0.90 for the CASI data set and 0.78 for MIMIC.

### Applied Data Sets

For the development and assessment of methodologies, most studies utilize a diverse range of data sets, as detailed in Table 5.

**Table 5:** Applied data sets for the creation and/or evaluation, and language of clinical narratives being processed, are listed.

Paper reference	Data set for creation or evaluation^a^	Language
Adams et al [22]	*MIMIC-III*^b^*,* University of Minnesota (CASI^c^)^d^, Columbia University intensive care unit/critical care unit reverse substitution^d,e^, MIMIC reverse substitution^e^	English
Avdic et al [23]	*Unstructured EHR*^f^*medical reports* and custom dictionary of medical (eg, diagnoses, medications, Latin terms) and nonmedical terms (stop words, proper nouns)	Serbian
Egorov and Funkner [24]	*Unstructured EHR medical recommendations*, Leo Tolstoy’s novel “War and Peace,” and an encyclopedic dictionary of medical Russian terms	Russian
Jaber and Martínez [30]	University of Minnesota (CASI)^d^	English
Jaber and Martínez [25]	University of Minnesota (CASI)^d^ *and unstructured EHR admission notes/inpatient consult notes/discharge summaries*	English
Luo et al [15]	*i2b2* ^g^ *2010*	English
Skreta et al [26]	*MIMIC-III*, University of Minnesota (CASI)^d^, and *i2b2 2010*	English
Grossman et al [16]	UMLS^h^-LRABR^i^, ADAM^i,j^, Berman’s abbreviations^i^, Wikipedia^i^, Vanderbilt University inventories from EHR from sign-out and discharge notes^d^, Stetson^d^, Columbia OBGYN^d^, and *MIMIC-III*	English
Grossman Liu et al [17]		
Wang et al [18]	Medical Subject Headings abbreviations via MEDLINE abstracts^i^, University of Minnesota (CASI)^d^, and Clinical Abbreviations from Vanderbilt University^d^	English
Link et al [27]	*Unstructured EHR clinical notes*	English
Khaleghi et al [28]	*Unstructured EHR surgical notes*	English
Kashyap et al [29]	*MIMIC-III*, PubMed Central, and University of Minnesota (CASI)^d^	English
Joopudi et al [19]	University of Minnesota (CASI)^d^ *and unstructured EHR longitudinal patient records*	English
Rajkomar et al [20]	University of Minnesota (CASI)^d^, *MIMIC-III*, synthetic snippets, *i2b2 2014*, Clinical Abbreviations from Vanderbilt University^d^, Sign-out note abbreviations^i^, Beth Israel Deaconess Medical Center abbreviations^i^, and Wikipedia^i^	English
Mykowiecka and Marciniak [31]	*Unstructured EHR clinical notes (interrogation, examination, and recommendations)*	Polish
Báez et al [21]	*Chilean Waiting List Corpus: unstructured EHR clinical notes (referrals)*	Spanish
Seneviratne et al [32]	University of Minnesota (CASI)^d^	English
Agrawal et al [33]	University of Minnesota (CASI)^d^ and MIMIC-III reverse substitution^e^	English

^a^Clinical narrative data sets are marked in *italics*.

^b^MIMIC: Medical Information Mart for Intensive Care.

^c^CASI: Clinical Abbreviation Sense Inventory.

^d^A data set that consists of a sense inventory in combination with short forms in context.

^e^Reverse substitution: replacement of long forms with their short forms and labeling it with the original target label.

^f^EHR: electronic health record.

^g^i2b2: Informatics for Integrating Biology and the Bedside.

^h^UMLS: Unified Medical Language System.

^i^Short-form–specific sense inventories.

^j^ADAM: Another Database of Abbreviations in MEDLINE.

Grossman et al [16] and Grossman Liu et al [17] utilized 9 data sets to create a comprehensive deep database of medical abbreviations. Similarly, Rajkomar et al [20] used 8 data sets for clinical abbreviation disambiguation. Additionally, 7 of 19 (37%) studies used unstructured EHRs from their institutions, which are not publicly accessible due to privacy concerns. Available clinical narratives constituted a significant portion of the listed data sets. Notably, 10 of 19 (53%) studies used the CASI [9] from the University of Minnesota, which includes a sense inventory, document-level metadata, and context information for ambiguous clinical abbreviations. The CASI data set incorporates several data sets that are also used independently by other studies reviewed here, including ADAM (Another Database of Abbreviations in MEDLINE) [63]; the UMLS Metathesaurus [64]; and Stedman’s Medical Abbreviations, Acronyms and Symbols [65]. Similarly, 6 of the 19 (32%) papers utilized the MIMIC-III [7], which is a deidentified intensive care data set covering over 40,000 patients. This data set includes intensive care notes, tests, orders, billing and code information, demographics, and reports for patients attended to by hospital staff between 2001 and 2012.

### Languages Under Investigation

Examining the language distribution of data sets used across the 14 eligible papers, 14 of the 19 (74%) data sets were in English. The remaining data sets were in other languages: Serbian [23], Russian [24], Polish [31], and Spanish [21]. Given that these are considered low-resource languages for clinical NLP, custom dictionaries and sense inventories for short-form content were necessary to aid in expansion and disambiguation. These inventories were then applied to process texts for the predetermined tasks of short-form identification, expansion, and disambiguation.

### NLP Recommendations

To assess the quality and reproducibility of the included papers, NLP recommendations across 7 categories were analyzed: source code availability, linking to external data sets, descriptions of internal data sets, application of performance metrics, provision of error analysis, inclusion of confusion matrices, and execution of external validation. These categories were inspired by the NLP recommendations from Kersloot et al [13], established for future studies during a systematic scoping review on NLP algorithms for mapping clinical text fragments onto ontology concepts. The classification of each included paper for short-form processing according to these categories is summarized in Table 6.

Only 3 of 19 (16%) papers met all the recommendations. By contrast, 16 of 19 (84%) only partially fulfilled the criteria for each category. Specifically, of the 19 papers, 10 (53%) did not provide the source code for their methodology, 2 (11%) did not link to the external data sets used, 15 (79%) did not include a confusion matrix for error analysis, 10 (53%) did not conduct an error analysis, and 9 (47%) did not perform external validation.

**Table 6:** Analysis and classification of included papers according to their fulfillment of natural language processing recommendations for identification, expansion, and disambiguation of short forms.^a^

Paper reference	Overall fulfillment	Source code	External data set linked	Internal data set described	Performance metrics	Error analysis	Confusion matrix	External validation
Adams et al [22]	Partially	Yes	Yes	Partially	Yes	No	No	Yes
Avdic et al [23]	Partially	No	No	Yes	Yes	Yes	No	No
Egorov and Funkner [24]	Partially	No	No	Partially	Yes	No	No	No
Jaber and Martínez [30]	Partially	No	Yes	N/A^b^	Yes	No	No	No
Jaber and Martínez [25]	Partially	No	Yes	Yes	Yes	Yes	No	No
Luo et al [15]	Yes	Yes	Yes	N/A	Yes	Yes	N/A	Yes
Skreta et al [26]	Yes	Yes	Yes	N/A	Yes	Yes	Yes	Yes
Grossman et al [16]	Partially	Yes	Yes	N/A	Yes	No	No	Yes
Grossman Liu et al [17]	Partially	Yes	Yes	N/A	Yes	No	No	Yes
Wang et al [18]	Partially	No	Yes	Yes	Yes	Yes	No	No
Link et al [27]	Yes	Yes	N/A	Yes	Yes	Yes	Yes	Yes
Khaleghi et al [28]	Partially	No	N/A	Yes	Yes	No	Partially	Yes
Kashyap et al [29]	Partially	No	Yes	Yes	Yes	No	No	Yes
Joopudi et al [19]	Partially	No	Yes	Yes	Yes	Yes	No	Yes
Rajkomar et al [20]	Partially	Yes	Yes	Yes	Yes	Yes	No	Yes
Mykowiecka and Marciniak [31]	Partially	No	N/A	Yes	Yes	Yes	No	No
Báez et al [21]	Partially	Yes	Yes	Yes	Yes	No	No	No
Seneviratne et al [32]	Partially	Yes	Yes	N/A	Yes	No	No	No
Agrawal et al [33]	Partially	No	Yes	N/A	Yes	No	No	No

^a^Four possible classifications: yes, no, partially, and N/A.

^b^N/A: not applicable.

## Discussion

### Principal Findings

Research on abbreviations has not uniformly covered all types, and methodologies have yielded varying results for different subtypes. This disparity is partly due to the limitations of existing short-form repositories, which often focus on specific types of abbreviations. For instance, the CASI from the University of Minnesota includes only acronyms and omits single-character abbreviations. Developing a comprehensive sense inventory for single-character abbreviations is challenging and may require utilizing openly available data sets and databases. The higher ambiguity associated with single-character abbreviations could also explain why they are frequently excluded from studies.

It appears that clinical narratives in different languages have varying distributions of short forms and their subtypes [4]. The included articles indicate that short forms can include the following subtypes: abbreviations, acronyms, abbreviations followed by a period, single-character abbreviations, among others. Our systematic scoping review found that all these subtypes were addressed in at least one of the articles reviewed. However, not all articles clearly specify the types of short-form content they processed. Nevertheless, it is often possible to infer the subtypes covered, based on the methods described or examples from the data sets.

The preemptive exclusion of certain types of short forms from data sets introduces a bias that becomes apparent only when the articles are carefully compared individually.

For identifying and expanding short forms in clinical narratives, the most commonly used approaches were rules, string similarity, and lookups in lexical resources. These methods have a low barrier to entry, being relatively easy to implement and test. By contrast, DL and ML approaches require significantly more resources, including graphics cards, high computing power, and large data sets. Our review indicates a strong preference for DL, particularly for disambiguation tasks, as it heavily relies on contextual detection.

### Limitations and Future Research Directions

A limitation of this systematic scoping review was the restriction to the past 5 publication years, which may have excluded important studies published before or after this period. This decision was driven by practical considerations, such as managing the volume of literature within the constraints of available personal resources.

Limitations in effectively processing all types of short forms are primarily due to the data sets and resources used to develop the processing methodologies. Key factors include data accessibility and the resources required for implementing short-form processing techniques. These limitations arise from the need for high-quality data collected from various sites or institutions for each data set language and the establishment of annotation workflows for creating comprehensive short-form sense inventories. These processes would enable the semantic recording of different documentation styles and varied contexts for each short form, thereby enhancing the processing algorithms to better recognize, expand, and disambiguate all types of abbreviations. Given the particularities of clinical language and short forms in each language, bridging the gap in processing between languages requires higher-quality resources. These resources should be generated and made accessible to the research community to improve cross-linguistic short-form processing [66].

Additionally, the articles examined in this scoping review only marginally reflect the impact of LLMs, such as those popularized by ChatGPT. Only 1 article in the review applied LLMs for short-form processing. This limited representation is partly due to the review’s timeframe, which concluded just a few months after the release of the GPT-3.5 model. Since then, numerous studies have utilized LLMs for clinical short-form processing tasks. However, these results offer only a snapshot amid the rapidly evolving technological landscape. Currently, LLMs appear to hold significant potential for clinical short-form processing, although their precision is still challenged by issues such as hallucinations, which are difficult to control. Additionally, many of the most effective models are proprietary and cloud-based, which limits their use for processing sensitive data. Therefore, the methodologies analyzed and discussed in this scoping review should not be considered obsolete. We plan to update this review once LLM technology has matured and a sufficient number of new studies—ideally utilizing the same data sets as those reported here—have been published.

### Conclusions

Short-form expressions, such as acronyms and other abbreviations, are distinctive elements found in narratives written by clinicians and stored in EHRs. To gain an overview of methods for processing these short-form expressions in clinical texts, we conducted a systematic scoping review of peer-reviewed articles. Our review found that classical ML and DL methodologies demonstrated the best performance for short-form disambiguation tasks, while rule-based and string similarity matching approaches were more commonly used for short-form identification and expansion. The methodologies applied to different short-form types and languages varied, and recommendations for NLP studies were only partially followed. Future research should focus on improving the quality and reproducibility of investigations by providing comprehensive details, including links to used resources and a more detailed description of the short-form content being studied.

## Appendix

Multimedia Appendix 1 PRISMA-ScR (Preferred Reporting Items for Systematic Reviews and Meta-Analyses-Scoping Review) checklist.

## Abbreviations

BERT: bidirectional encoder representations from transformers
CARD: Clinical Abbreviation Recognition and Disambiguation
CASI: Clinical Abbreviation Sense Inventory
CLASSE GATOR: Clinical Acronym Sense Disambiguator
CNN: convolutional neural network
CUIMC: Columbia University Irving Medical Center
DL: deep learning
EBMR: Evidence-Based Medicine Reviews
EHR: electronic health record
ELECTRA: Efficiently Learning an Encoder that Classifies Token Replacements Accurately
GPT: generative pretrained transformer
i2b2: Informatics for Integrating Biology and the Bedside
LLM: large language model
MeSH: Medical Subject Headings
MIMIC: Medical Information Mart for Intensive Care
ML: machine learning
NLP: natural language processing
PRISMA: Preferred Reporting Items for Systematic Reviews and Meta-Analyses
SVM: support vector machine
UMLS: Unified Medical Language System
